# ISG15 driven cellular responses to virus infection

**DOI:** 10.1042/BST20220839

**Published:** 2022-11-23

**Authors:** Deeksha Munnur, Adrianna Banducci-Karp, Sumana Sanyal

**Affiliations:** Sir William Dunn School of Pathology, University of Oxford, South Parks Road, Oxford OX1 3RE, U.K.

**Keywords:** immune response, ISG15, post translational modifications, RNA viruses, virus–host interactions

## Abstract

One of the hallmarks of antiviral responses to infection is the production of interferons and subsequently of interferon stimulated genes. Interferon stimulated gene 15 (ISG15) is among the earliest and most abundant proteins induced upon interferon signalling, encompassing versatile functions in host immunity. ISG15 is a ubiquitin like modifier that can be conjugated to substrates in a process analogous to ubiquitylation and referred to as ISGylation. The free unconjugated form can either exist intracellularly or be secreted to function as a cytokine. Interestingly, ISG15 has been reported to be both advantageous and detrimental to the development of immunopathology during infection. This review describes recent findings on the role of ISG15 in antiviral responses in human infection models, with a particular emphasis on autophagy, inflammatory responses and cellular metabolism combined with viral strategies of counteracting them. The field of ISGylation has steadily gained momentum; however much of the previous studies of virus infections conducted in mouse models are in sharp contrast with recent findings in human cells, underscoring the need to summarise our current understanding of its potential antiviral function in humans and identify knowledge gaps which need to be addressed in future studies.

## Introduction

Upon infection, viral pathogens are detected by an array of innate immune sensors distributed across various intracellular compartments. Depending on the type of virus and their genetic makeup (e.g. DNA, RNA) these pathogen recognition receptors are activated to initiate a signalling cascade culminating in the production of a range of cytokines [1,2]. Among others, interferons are secreted from these infected cells and bind to interferon receptors that trigger a JAK/STAT-dependent signalling cascade to produce hundreds of interferon-stimulated genes (ISGs) [3,4], ISG15 being one of them. It is one of the most abundant and early response genes with versatile functions in restricting different stages in the viral life cycle, either by directly modifying and inhibiting viral factors, or by altering the host landscape and limiting resources available for the progression of the viral lifecycle. In addition, ISG15 itself in its unconjugated form can be secreted both by myeloid cells and lymphocytes to potentially recruit and activate immune cells to sites of infection [5].

**Figure 1.**
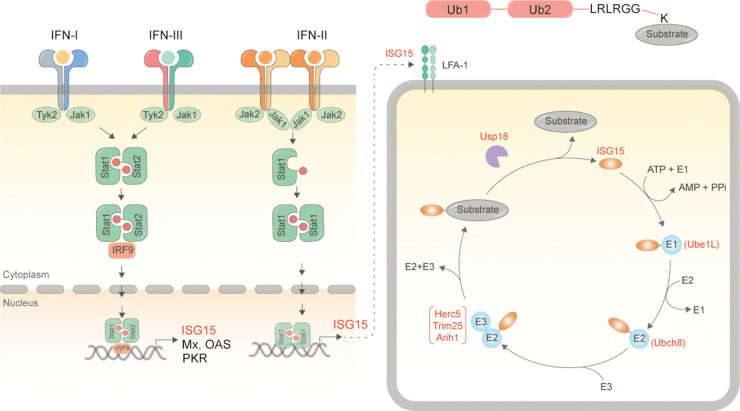
Schematic for synthesis of ISG15 and ISGylated proteins. Binding of interferons to interferon receptors (type I, II, III) as a consequence of viral infection results in activation of the JAK/STAT signalling cascades [3,4]. Type I and III interferons bind to heterodimeric IFNAR1/IFNAR2 whereas type II binds to IFNAR1/IL-10R, triggering phosphorylation of Stat1/Stat2 complexes. The trimeric complex with IRF9 traffics to the nucleus to bind interferon response elements to promote transcription of interferon stimulated genes. ISG15 is one of the early and most abundant ISGs produced in IFN-stimulated cells. ISG15 can either be secreted out from these cells [5] or be conjugated to substrates via the sequential activity of Ube1L [7], Ubch8 [8] and Herc5 [9,10]/Trim25 [11,12]/ARIH1[13,14] enzymes. Modification is reversed by the activity of the de-ISGylating enzyme Usp18 [15], while free ISG15 can be taken up by binding to LFA-1 [85].

ISG15 is initially synthesised as a 17 kDa precursor protein and processed into a 15 kDa form [6]. While it exists at low levels under normal physiological conditions, its expression increases by at least an order of magnitude upon binding of interferon regulatory factors (IRFs) to interferon response element (IRSE) containing promoters [6]. This can occur either upon stimulation with α and β interferons or infection with viral and bacterial pathogens. ISGylation occurs via an enzymatic cascade that is analogous to ubiquitylation: an E1 enzyme (Ube1L [7]) forms a thioester intermediate at the C-terminal group of ISG15 in an ATP dependent manner, and transfers activated ISG15 to the E2 enzyme (Ubch8 [8]). The E2 enzyme transfers ISG15 to an E3 ligase (e.g. Herc5 [9,10], Trim25 [11,12], ARIH1 [13,14]), which in turn executes the final step of covalently attaching ISG15 to specific lysine residues of substrates. Like ubiquitylation, ISGylation too is reversible and can be hydrolysed by Usp18 [15], a cysteine protease. Unlike ubiquitylation however, ISG15 is not known to form polymeric chains (Figure 1).

The importance of the ISGylation pathway is underscored by consequences of mutations in either the *Isg15* gene or those encoding associated enzymes. Frameshift mutations in the ubiquitin like domain of ISG15 have been linked with recurrent skin lesions, cerebral calcification and lung disease [16–18]. Autosomal recessive complete Usp18 deficiency is lethal in infancy due to hyperinflammation and excessive interferon signalling [19]. More recently a homozygous mutation in Usp18 was linked to insufficient down-regulation of interferon signalling, defective IL-12 and IL-23 induction and increased susceptibility to mycobacterial disease [20].

## ISG15-mediated host responses during viral infection

*In vivo* mouse models have yielded variable and often contradictory results on the role of ISGylation in antiviral responses. For example, initial studies with Usp18^−/−^ mice underscored the importance of ISGylation in antiviral responses [21]. Usp18^−/−^ mice were protected from lethal LCMV and VSV infection, displaying increased ISG15 conjugates, diminished virus replication and virus-specific T cell responses, implicating ISGylation as the basis for this phenotype [21]. This was in sharp contrast with studies with ISG15^−/−^ mice, which displayed no effect on interferon signalling, or resistance to LCMV and VSV infection [22]. These findings have been validated in later studies, which also displayed normal levels of virus infection in Ube1L^−/−^ mice, but reduced replication in Usp18^−/−^ mice [23,24]. Much of these confounding results stems from the dual role of Usp18: on the one hand as a de-ISGylase, and on the other as a negative regulator of interferon signalling. Generation of the catalytically dead Usp18 knock-in mice inactivating solely its de-ISGylating activity [25] has shed light on the multiple functions of Usp18 as summarised in other excellent reviews [26,27].

**Figure 2.**
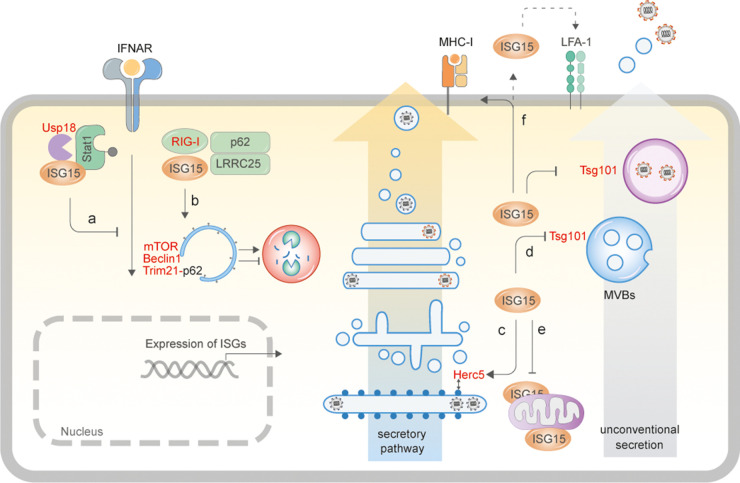
ISG15-driven cellular responses. (**a**) Human ISG15 binds to Usp18 and prevents its ubiquitylation and proteasomal degradation [17,55]. Usp18 in turn binds to Stat2 and down-regulates IFNAR2, thereby suppressing IFN-signalling. In the absence of ISG15, Usp18 is degraded, resulting in prolonged IFN-I signalling and inflammatory responses [17,55]. (**b**) ISG15 binds to RIG-I-LRRC25-p62 to induce their autophagosomal degradation and prevent excessive immune activation [52], and has also been shown to trigger autophagy by modifying mTOR in HEK293T cells [54], but equally is able to block autophagic flux by modifying Beclin1 [49] and Trim21 [51]. (**c**) ISG15 modifies newly synthesised secretory proteins via Herc5 association with polyribosomes [35]. In infected cells, this phenomenon results primarily in ISGylation of viral capsids leading to inhibition of virus assembly. ISGylation of proteins of the secretory pathway can potentially increase secretion of cytokines [42] and MHC-I presentation [43]. (**d**) ISGylation of components of the multivesicular body results in inhibition of exosomal release, and transport and secretion of virus proteins/particles via unconventional secretory processes [32,41]. (**e**) ISGylation of mitochondrial components can result in dysregulation of metabolic processes [53,74]. (**f**) Monomeric, free ISG15 can be secreted out of cells [69] to function as a cytokine by binding to LFA-1 expressing cells [85]. Verified interaction or modification of proteins by ISG15 is depicted in red.

While ISG15 has appeared to be an antiviral effector from studies in mouse models, data from ISG15-deficient patients has shown otherwise. For example ISG15-deficient patients have been found to be more susceptible to mycobacterial infections; however, they display increased resistance to viral infections on account of sustained IFN signalling and presence of ISGs [16]. The following sections therefore specifically focus on current data on the role of ISG15 available from human cellular models as illustrated (Figure 2).

### Direct impact of ISG15 on virus replication and spread

Several human cell-based studies have shown that ISG15 conjugation or binding to host and viral factors can directly inhibit the lifecycle of several viruses. The first evidence came from influenza virus infections, where ISG15 was shown to bind the viral non-structural protein 1 (NS1) of influenza B [28]. Subsequent studies have shown that ISGylation of influenza A viral NP even at low levels results in impaired packaging of viral RNPs [29]. ISG15 can modify and inhibit a range of viral factors, thereby impairing the viral lifecycle. These include influenza A [30–32], retroviruses [33], Hepatitis B and C, vaccinia, Zika and Ebola [34], among others. The broad substrate specificity of Herc5 and its physical association with polyribosomes has prompted a model in which newly synthesised viral proteins in infected cells are ISG15-modified to impair their function [35]. The impact of ISG15 conjugation or binding to viral factors to inhibit infection has been extensively reviewed elsewhere [36–38], which points toward its antiviral nature. Interestingly, more recent studies have also revealed a pro-viral function of ISG15 in replication of Hepatitis B [39] and C viruses [40]. These anecdotal reports indicate that co-evolution of viruses with their host may have enabled these pathogens to exploit ISGylation to their own benefit.

Besides affecting virus replication by directly interacting with or modifying viral factors, ISGylation was also found to inhibit virus release by modifying components of the multivesicular pathway. Experiments performed in influenza virus infected semi-permeabilised cells revealed Tsg101 as one such component modified by ISG15 upon immune activation, resulting in impaired transport of virus glycoproteins and budding of viral progenies [32]. These studies were further extended to show that ISGylation of exosomal components triggered their aggregation followed by lysosomal degradation, thus impeding exosomal release [41]. The effect of ISGylation on secretory processes is less than straightforward. While exosomal transport is inhibited by ISGylation, conventional secretory processes are enhanced. ISGylation of proteins of the secretory pathway was found to increase cytokine secretion in a Listeria infection model [42]. Similarly, ISGylation has also been shown to increase antigen presentation on major histocompatibility complex-I at the cell surface [43,44], implicating it in potentially facilitating canonical secretory processes under certain conditions.

### Regulation of autophagy

Autophagy functions as a cell intrinsic pathway to dispose intracellular pathogens - a process often manipulated by viruses to their own advantage [45–48]. ISGylation has been reported to regulate autophagy in several different ways during virus infection. ISGylation of Beclin1 was shown to inhibit PIK3C3 kinase activity, thereby suppressing autophagy [49]. Lysine residues 117 and 263 in Beclin1 were identified as primary ISGylation sites, which in turn competed with K63-linked ubiquitylation. Type-I interferon signalling was therefore found to inhibit autophagosomal turnover of EGFR [49]. These findings were further consolidated in recent reports, which identified Respiratory syncytial virus (RSV) NS2 as an antagonist of this pathway. NS2 was found to promote pro-viral autophagy by stabilising Beclin1 and preventing its ISGylation [50]. These data on the negative regulation of autophagy are in line with previous studies on IFNβ-induced ISGylation of Trim21. Modified Trim21 with enhanced E3-ligase activity resulted in Trim21-mediated p62 ubiquitylation, preventing its oligomerisation and recruitment to autophagosomes, thereby inhibiting this process [51]. On the other hand, ISG15 has been reported to promote selective autophagic degradation of RIG-I [52] as well as of mitochondria (mitophagy) [53]. In vaccinia infected bone marrow derived macrophages, ISG15 deficiency resulted in dampened mitophagy. This however, was hypothesised to occur on account of altered mitochondrial metabolism rather than via a direct impact on autophagosomal components [53]. In vivo ISGylome studies in murine models following bacterial infection has revealed several proteins such as mTOR, WIPI2, AMBRA1 and RAB7 that are ISG15 modified, resulting in increased autophagy [54]. These contrasting data therefore underscore differences that might exist in human versus murine models of infection and/or of alterations incurred upon viral versus bacterial infection [55].

### ISG15 and lipid droplets

An interesting recent study designed to capture ISG15 interactors point toward concentration of ISGylated proteins on lipid droplets [56]. One of the candidates identified was the ring finger E3-ligase Rnf213, mutations in which are associated with Moya Moya disease [57]. Rnf213 was found to be an ISG15 interactor and also function as a sensor for ISGylated proteins. Interferon induction upon viral (e.g. Herpes simplex, RSV and coxsackie viruses), or bacterial infection (e.g. *Listeria monocytogenes*) resulted in oligomerisation of Rnf213 on lipid droplets [56,58]. These findings are particularly interesting in light of emerging concepts on the antiviral functions of lipid droplets. Proteomic analyses of lipid droplets from LPS treated cells have revealed accumulation of several innate immunity-associated factors (e.g. IFNγ inducible GTPases, viperin, IFI47) that are known to be antiviral in nature [59]. Altered morphology and composition of lipid droplets in interferon treated cells have also been observed [60]. How these interferon-stimulated lipid droplets contribute to virus restriction are currently under investigation [61].

### Modulation of innate immunity and inflammation

An emerging theme from recent studies on ISG15 is its impact on innate immune responses to infection, both in its free and conjugated forms. Initial proteomics studies conducted with expression of tagged ISG15 revealed many substrates that participate in innate immune signalling cascades. These include STAT1 [62], JAK1 [62], RIG-I [63], IFIT1, PKR, MxA, IRF3 [64]. STAT1 is ISGylated in human cells, which was found to maintain the required levels of phosphorylated, activated STAT1 and downstream signalling. This modification therefore results in a more robust interferon response that limits viral replication [65]. Similarly, ISGylation of IRF3 results in its stabilisation and therefore promotes downstream signalling [64]. Recent case studies of individuals with inherited ISG15 deficiency who display signs of autoinflammation support this immunomodulatory function of ISG15 in the IFN-signalling cascade [16,17]. Interestingly, while previous studies had shown ISGylation to inhibit HIV budding via Gag modification in infected monocytes and macrophages [66], a recent report demonstrated that fibroblasts from *ISG15*-deficient patients were more resistant to HIV infection, underscoring the confounding effects of ISGylation on individual stages of the viral lifecycle versus host immune responses to them [67].

Several studies over the past few years have reported on altered secretion of cytokines emanating from ISG15 deficiency. For instance, monogenic type I interferonopathies are a group of autoinflammatory and autoimmune disorders characterised by persistently elevated levels of type I interferons (IFN-I). The underlying molecular mechanisms include enhanced IFN-I gene transcription due to gain of function of STAT2 and defective negative regulation of IFN-I as a result of either *ISG15* or *USP18* deficiency [68]. Clinical findings of *ISG15* deficiency are consistent with chronic hyperinflammation, originating from innate immune cells such as macrophages. ISG15^−/−^ fibroblasts, keratinocytes and iPSC-derived endothelial cells also display hyperinflammation, consistent with increased production of proinflammatory cytokines [18]. Besides the effect of conjugated ISG15 on cytokine production, free extracellular ISG15 has also been shown to possess immunomodulatory properties [69]. Most prominently, secreted vesicles collected from *M Tb* infected cells were found to contain free ISG15. When these were added to naïve monocytes, it triggered production of proinflammatory cytokines such as IL-8, MIP-1α and IP-10 [70]. Subsequent studies have shown that secreted ISG15 can also stimulate production of the anti-inflammatory cytokine IL-10 [71].

### ISG15-dependent metabolic reprogramming and mitochondrial function

We recently reported that in virus-infected macrophages enzymes of the glycolytic pathway and mitochondrial enzymes are major targets of ISGylation [72], and in turn are de-ISGylated by viral proteases. These findings are in line with several other studies, for example in adipocytes, which showed that ISG15-dependent reprogramming of the glycolytic pathway underlies suppression of genes involved in thermogenesis [73]. Similarly, HeLa cells infected with Coxsackievirus show increased glucose consumption on account of increased energy demands during virus replication; elevation in glycolysis is further increased in ISG15^−/−^ cells, supporting its role in suppressing glycolysis [74]. Whether it is a direct effect of ISGylation of glycolytic enzymes needs further validation.

In a separate study with induced pluripotent stem cell-derived macrophages and endothelial cells as models, *ISG15*^−/−^ macrophages displayed the expected hyperinflammatory responses, but normal phagocytic function [75]. Their pathology included a range of dysregulated processes such as increased apoptosis/pyroptosis, oxidative stress and glycolysis, but decreased oxidative phosphorylation, β-oxidation, and NAD(P)H-dependent oxidoreductase activity [75]. *ISG15*^−/−^ cells also displayed defective expression of genes involved in mitochondrial biogenesis and respiratory chain complexes II–V, resulting in diminished mitochondrial respiration. This phenotype was rescued upon reconstitution with wild-type ISG15, but only partially by a conjugation-deficient variant, suggesting that at least some of these phenotypes were driven by ISGylation to cellular targets [75]. Pharmacological treatment with itaconate and its derivatives, and the JAK1/2 inhibitor ruxolitinib could also reduce inflammatory responses, cell death, and oxidative stress [75].

Some of these data are in agreement with previous findings with vaccinia virus infection models in peritoneal macrophages. ISG15^−/−^ macrophages were found to exert reduced activity, phagocytic capacity and programmed cell death upon vaccinia virus infection. Peritoneal macrophages from ISG15-deficient mice too were unable to phagocytose infected cells nor block virus infection in co-cultures with infected fibroblasts [76]. Further studies revealed that ISG15 regulated mitochondrial function in macrophages. Mitochondrial respiration and production of ATP and ROS were found to be impaired in ISG15^−/−^ bone marrow derived macrophages upon IFN treatment, rendering them more susceptible to vaccinia virus infection [53]. Collectively, these findings expand the repertoire of ISG15-mediated cellular processes and reveal the importance of ISG15 in regulating cellular metabolism, oxidative stress, and mitochondrial function.

## Viral strategies of subverting ISG15-dependent responses

While cells mount an ISG15-dependent response to trigger defences against viral infection, many viruses have in turn co-evolved strategies to counter them. Particularly interesting in this regard are viral proteases that are able to function as de-ISGylases and remove ISG15 modifications from substrates to their own advantage. OTU-domain containing proteases expressed by several RNA viruses e.g nairoviruses, arteriviruses, are able to hydrolyse both ubiquitin and ISG15 modifications (unlike their mammalian counterparts [77]), with broad substrate specificity, thereby suppressing antiviral responses [78,79]. Other RNA viruses (e.g. coronaviruses, foot and mouth disease virus) encode papain like proteases that are equipped with de-ISGylating activities [72,80,81]. In a separate strategy, Influenza B virus NS1 sequesters ISGylated proteins by non-covalently binding to ISG15 [29]. While the targets of viral de-ISGylating enzymes have not been systematically identified, current evidence indicates that many of them are components of immune signalling processes (e.g. IRF3 [82], MDA5 [83]) and enzymes of the glycolytic pathway [72]. Besides their biological function, viral proteases have also served as critical experimental tools to provide fundamental insights into ISGylation [84].

An obvious consequence of de-ISGylation activity is an increased unconjugated, monomeric form of ISG15, which can either exist intracellularly, or be secreted out of cells. Early studies had indicated that extracellular ISG15 functioned as a signalling molecule to trigger release of IFNγ from lymphocytes [5]. Support of these data was acquired later with blood leukocytes from patients with inherited ISG15-deficiency receiving the BCG vaccine, who were impaired in IFNγ production compared with control samples [17]. The receptor for extracellular ISG15 was identified later as LFA-1 integrin [85]. An elegant ISG15-reporter based assay to study the role of extracellular ISG15 revealed that viral de-ISGylases triggered secretion of free ISG15 into the extracellular space where it induced NK cells and T lymphocytes to produce pro-inflammatory cytokines [81]. Combined with previous observations that ISG15-deficiency results in hyper inflammatory responses, e.g. IL-6, IL-8, IL-1β production on account of prolonged IFN-I signalling [16], the activity of viral proteases would imply a dual mechanism of triggering hyper inflammation: first via impaired IFN down-regulation and second, via extracellular ISG15 activity. This was empirically tested in iPSC-derived macrophages expressing either the wild-type or the catalytically dead version of SARS-CoV-2 PLpro. Many of the inflammatory signatures observed in infection with live virus could be recapitulated in the macrophages expressing the wild-type but not the catalytically dead PLpro, indicating that its de-ISGylating activity is sufficient to drive such responses [72,86].

Proteomics analyses revealed glycolytic enzymes as major targets of PLpro. In addition, components of purine metabolism and immune signalling were found to be PLpro substrates. Metabolic reprogramming by altering glycolysis has been consistently observed in different experimental and disease models. Reduced thermogenic gene expression and oxygen consumption in adipose tissue resulted from ISG15-mediated inhibition of glycolytic enzymes [73]. iPSC-derived ISG15^−/−^ macrophages display a complex pathological phenotype of increased glycolysis, oxidative stress and apoptosis, but decreased oxidative phosphorylation, β-oxidation and NADH-dependent oxidoreductase activity, underscoring its importance in mitochondrial function [75].

## Summary

While ISGylation has been studied extensively in the context of viral infections to identify potential mechanisms of virus restriction, emerging data from several independent studies indicate that a significant number of substrates are enzymes of various metabolic processes. This is particularly interesting since ISG15-driven metabolic reprogramming may explain many of the phenotypes observed during stress conditions, including those generated during either viral or bacterial infections. Given the link between cellular metabolic states and immune responses, it may also be a major determinant of inflammatory responses accompanying infection or other stress conditions such as DNA damage [87].

Despite increased momentum in ISG15 research, several knowledge gaps remain. For example, delineating the contribution of free versus conjugated ISG15 in pathological conditions is one such area. Till date, three E3-ligases have been identified for ISG15 conjugation to substrates [9–13]. Of these, while Herc5 is a dedicated ISG15 E3-ligase, Trim25 and ARIH1 can equally function as ubiquitin E3-ligases, begging the question of how many of the ∼700 E3-ligases are able to carry out ISGylation. In the same vein, distinguishing between the mechanism and consequences of co-translational (as seen for Herc5-mediated modification of newly synthesised proteins [35]) versus post-translational ISGylation is equally important. Although some experimental evidence indicates its unconventional mode of secretion, it will be worth investigating whether multiple routes exist for ISG15 secretion, and if receptors (besides LFA-1) can function for the uptake of free ISG15. Furthermore, while observations have been made on mixed chains of ubiquitin and ISG15, their physiological relevance is far less clear [88]. The recent discovery of ISG15 in bacterial immune defence may offer a simpler model system to interrogate some of these mechanisms [89]. Investigations on these aspects will not only provide fundamental insights, but may also prove useful in developing strategies to combat hyper-inflammation accompanying infection or autoimmune disorders. Towards this end, developing strategies to therapeutically target ISG15 or ISGylation, either via small molecule inhibitors, specific antibodies, or by chemical biology- based strategies akin to PROTACS would prove to be immensely beneficial [90].

## Perspectives

- ISG15 has emerged as an abundant and versatile post-translational modification not only in the context of infection, but also in a range of stress conditions such as metabolic disorders, DNA damage, autoimmunity and inflammatory diseases. Understanding the fundamental principles of ISGylation therefore has far-reaching implications.
- Our current understanding specifically on the antiviral role of ISG15 indicates that it does not directly inhibit the viral lifecycle. Instead it is part of the host response that alters the immuno-metabolic network to restrict availability of resources necessary for viral amplification, thereby limiting virus spread. Further studies in simpler organisms such as bacterial defence against phages may shed more light on the fundamental and evolutionarily conserved role of ISG15.
- Several questions remain outstanding in this field, not the least of which is whether ISG15 can be targeted for therapeutic applications. Future studies on developing small molecule or antibody-based inhibitors to ISG15 and its associated enzymes will not only address the potential for therapy, but also resolve the confounding effects of free versus conjugated forms of ISG15.

## Data Availability

Data sharing is not applicable to this article as no datasets were generated or analysed in the current study.

## Open Access

Open access for this article was enabled by the participation of University of Oxford in an all-inclusive *Read & Publish* agreement with Portland Press and the Biochemical Society under a transformative agreement with JISC.

## Abbreviations

IFN: interferons
IRFs: interferon regulatory factors
ISG15: interferon stimulated gene 15
NS1: non-structural protein 1
RSV: respiratory syncytial virus
